# Prevention of overweight and obesity in a Norwegian public health care context: a mixed-methods study

**DOI:** 10.1186/s12889-021-11096-x

**Published:** 2021-05-26

**Authors:** T. Westergren, L. Fegran, A. Jørstad Antonsen, H. Timenes Mikkelsen, C. B. Hennig, U. M. Stamnes Köpp

**Affiliations:** 1 Department of Health and Nursing Science, Faculty of Health and Sports Sciences, University of Agder, Postboks 422, 4604 Kristiansand, Norway; 2 Department of Children and Adolescents, Southern Norway Hospital, Kristiansand, Norway

**Keywords:** Overweight, Pediatric obesity, Preschool children, Child health, Prevention, Primary health care, Public health nurse

## Abstract

**Background:**

Greater understanding about the prevention and treatment of overweight and obesity in preschool children within public health care is needed. This study assessed the impact of The First Steps module in routine primary health care including mapping of height/weight and diet followed by parental counselling of healthy habits on overweight and obesity in children aged 2 to 7 years. Further, we explored the experiences of public health nurses (PHNs) with the module.

**Methods:**

Body weight and height obtained in 2014 and 2016 were extracted retrospectively for 676 children from the health records of children at 2, 4, or 6 years of age in five child health centers in Southern Norway. Sex- and age-adjusted body mass index (BMI) *z*-scores and weight status classifications were calculated according to the International Obesity Task Force reference values. Impact was assessed as change in mean BMI *z*-scores for children with under-, normal-, and overweight, respectively, and as proportion of children with overweight and obesity. In focus groups, PHNs described their experiences with the practical application of the module. Focus group transcripts were analyzed using Braun and Clarke’s thematic analysis.

**Results:**

Mean BMI *z*-scores decreased from 2014 to 2016 in overweight children (− 0.26) and increased in children with under- (0.63) and normal weight (0.06), whereas the proportion of children with overweight and obesity was stable. PHNs believed that the module provides them with new tools that are useful for addressing the intricacies of childhood obesity. They described counseling sessions with families as “moving upstream in a river” and that overweight and obesity may be one of many complex challenges for these families.

**Conclusions:**

Mean BMI *z*-score decreased in children with overweight during the 2 years after initiation of The First Steps module. PHNs considered the module as useful for addressing children’s overweight and obesity, which was perceived as one of several complex challenges for most of these families. Specialist and evidence-based support is needed to address overweight and obesity in children in primary care. Further research should focus on integrating the issues relating to overweight and obesity within other family problems.

**Supplementary Information:**

The online version contains supplementary material available at 10.1186/s12889-021-11096-x.

## Background

Early childhood overweight and obesity predicts later childhood, adolescent, and adult overweight and obesity [1, 2]. Childhood obesity represents a major risk factor for needing the disability pension, premature mortality, and the development of chronic conditions [3, 4]. Childhood obesity is also associated with social and psychological challenges as well as impaired health-related quality of life [5]. There are limited data about the prevalence of overweight and obesity in preschool children, but the Norwegian Bergen Growth study from 2010 reported that prevalence of overweight including obesity was 12.7% and the prevalence of obesity was 1.4% in the youngest age group (2–5 years) [6]. According to the International Obesity Task Force (IOTF), overweight is defined as an age- and sex-adjusted body mass index (BMI) of 25 kg/m^2^, obesity as 30 kg/m^2^, and severe obesity as 35 kg/m^2^ [7].

Evidence-based prevention of obesity in preschool children is needed [8–10], and includes a multicomponent treatment that combines physical activity and dietary behavior, rewards for children, and modeling through peers, parents, and teachers [11, 12]. Multicomponent treatment of obesity requires multidisciplinary and intensive behavioral programs that target the caregiver’s perception of a child’s weight and motivation for change, recruitment within routine health care, and the inclusion of feasible and acceptable elements shaped according to family resources and socioeconomic status [13]. Caregivers’ ability to recognize their child’s overweight may be poor. Júlíusson et al. reported that 90% of parents with an overweight preschool child perceive the child as being of normal weight [14]. Moreover, the prevalence of overweight and obesity is associated with lower parental educational level [6, 15].

In a qualitative meta-synthesis of health-care professionals’ experiences addressing overweight in children, Bradbury et al. [16] reported that parents who are overweight and unmotivated to change, who have a complex family situation, and who deny the situation were more challenging for health-care professionals to work with [16]. Addressing the issue of overweight in children requires health-care professionals to have sufficient confidence in their own competence to ensure effective interactions with parents and for parents to feel comfortable seeking assistance. Such interactions should involve the use of effective assessment tools and language about overweight, regular follow-up, and a family-centered health-promoting approach [16].

Norway has issued evidence-based national guidelines for preventing and treating overweight and obesity in children within child health centers using stepwise interventions related to severity of the problem [17, 18]. In the current study, we have advanced these guidelines in collaboration between primary and specialist health care, naming this efforts as The First Steps module.

The aims of this study were (a) to investigate the impact of The First Steps overweight and obesity prevention and treatment module in routine primary health care including mapping of height/weight and diet followed by parental counselling of healthy habits in children aged 2 to 7 years, and (b) to explore public health nurses’ (PHNs’) experiences with the module.

## Methods

### Study design

This study was an ecological mixed-methods triangular design. Height and weight data for 2 years were collected retrospectively from all patient records in the collaborating municipalities. Next, this data was used to evaluate the impact of the First Steps advanced routine on overweight and obesity prevention and treatment. Qualitative focus groups with PHNs focused on their experiences with the module.

### Use of the first steps module in child health centers

The First Steps module was developed in response to a request from PHNs in five Southern Norway child health centers and according to the national guidelines [17, 18]. The module was first applied in January 2015. PHNs were instructed to act according to the national cutoffs for the different levels of interventions, and if a child’s weight increased rapidly or crossed upwards of three lines on the weight-for-height growth chart.

**Fig. 1:**
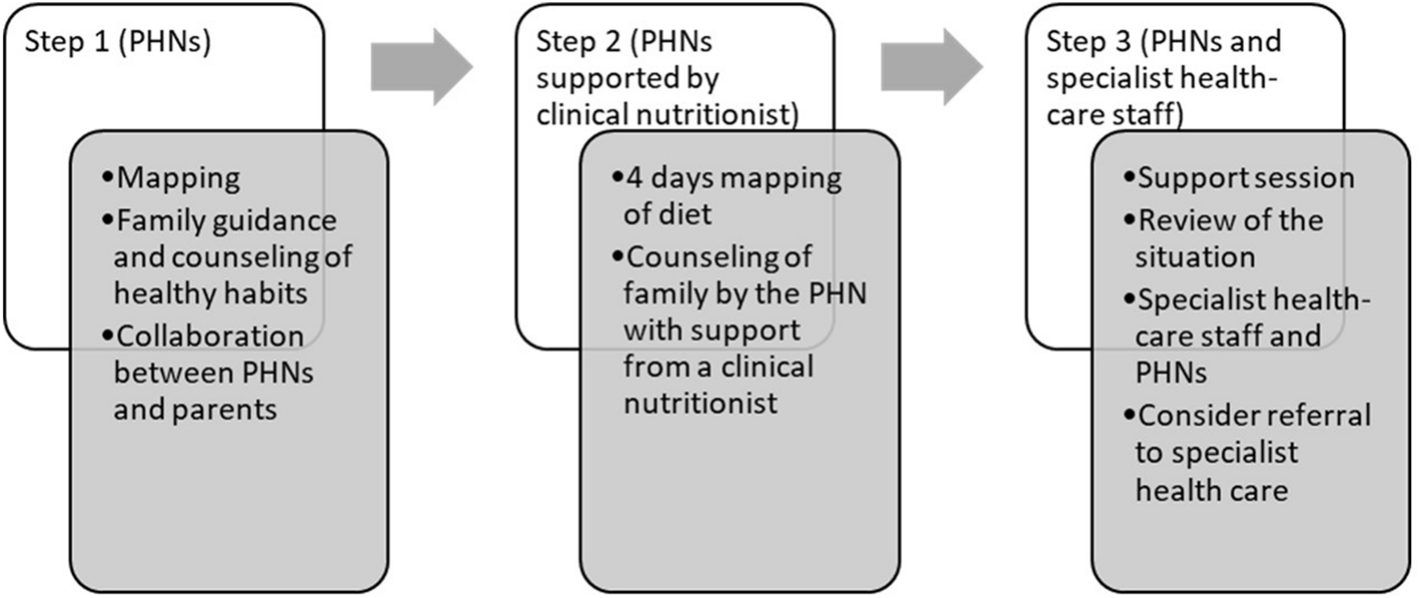
The First Steps module for child health centers targeting early overweight and obesity treatment according to the indicators of overweight/obesity contained in the national guidelines [17, 18], IOTF cutoffs [7], and/or crossing upwards of three lines in the WHO weight-for-height growth charts

In this module, which is briefly illustrated in Fig. 1, step 1 included mapping the child’s diet followed by counseling sessions with parents. This step focused on providing guidance and counseling about healthy habits and according to official health recommendations [18]. If the child’s weight gain continued, step 2 offered a 4-day mapping of the child’s eating behavior and dietary intake. The parents were responsible for creating the dietary schedule, which was evaluated by a clinical nutritionist who also provided written and specific advice to the parents that was communicated by the PHN. If the weight gain continued, step 3 offered families a counseling session led by a pediatrician and/or a clinical nutritionist from specialist health care. In this session, the needs of the family with a child with overweight were discussed and the PHN received additional guidance from the pediatrician and the clinical nutritionist about how to approach the family. Referral to specialist health care was considered at this stage if the child was obese.

In addition to the counseling sessions, a pediatrician and clinical nutritionist from specialist health care and PHNs from different municipalities participated in educational meetings. An information binder that included photographs of healthy food choices and written nutrition information was developed for use in counseling sessions with families.

### Participants and analysis of weight data

In 2014 and 2016, body weight and height were measured in all children by PHNs during ordinary routine follow-up (children aged 2, 4, and 6 years) for 773 and 919 children, respectively, and 616 children at two occasions. All children lived in the Southern Norwegian region. For clinical purposes, height and weight were plotted in growth charts that are based on national guidelines [17] and the WHO reference values [19, 20] for preschool children. Children were categorized as being of underweight, normal weight, overweight, or obese according to the sex- and age-adjusted IOTF reference values designed to cross ≤18.5, ≥ 25, and ≥ 30 at age 18, respectively [7]. Additionally, for research purposes, height and weight data were collected retrospectively from the patient records. Sex- and age-adjusted BMI *z*-scores were calculated according to the IOTF reference values [7] using the LMSGrowth Microsoft Excel plug-in [21]. For comparison, prevalence and sex- and age-adjusted BMI *z*-scores were also calculated using reference values and SPSS Statistics syntax provided online by the WHO [22, 23] for age 2–5 years and > 5 years, respectively. The impact of the advanced routine interventions was evaluated by change in mean BMI *z*-scores for children with under-, normal- and overweight, respectively. Stratified multiple linear regression analyses using change in BMI *z*-scores as the dependent variable with age, sex, and baseline BMI *z*-score as independent variables were conducted to evaluate confounding factors. Preliminary analysis was conducted to assess no violation of the underlying assumptions. McNemar’s test was used to evaluate changes in weight categories from overweight and obesity to normal weight and from obesity to overweight or normal weight between the two measurement times.

### Focus group participa2nts and procedures

Focus groups were conducted according to Krueger and Casey [24] to obtain in-depth insights and to elaborate on the PHNs’ views and experiences according to the study purpose. Twenty PHNs employed at five different child health centers in the region were invited to participate, and 11 (55%) agreed to participate. All five municipalities were represented by at least one PHN, and all had experience using The First Steps module from its initiation in January 2015 until December 31, 2016.

Two focus groups of five and six PHNs in each group were conducted in June and November 2017. The focus groups were conducted during working hours in conference rooms in the child health centers where the PHNs and the moderators could talk undisturbed.

The focus groups followed a semi-structured guide with open-ended questions focusing on PHNs’ experiences using The First Steps module. The main issues discussed during the focus groups were PHNs’ experiences using the module for the follow-up of children with obesity, indications used to identify follow-up needs, and collaborations between PHNs and between PHNs and the pediatrician and clinical nutritionist from the specialist health care. The PHNs in the groups were encouraged to speak freely and were given the opportunity to highlight other issues they perceived as relevant to the topic. During the focus groups, the moderators ensured that their perception of what was said and the PHNs’ own understanding were consistent through follow-up questions and mirroring, especially when unclear or implicit descriptions emerged. The focus group interviews lasted 1 h and 25 min (focus group 1) and 1 h and 18 min (focus group 2), and were audio-recorded and transcribed verbatim.

### Qualitative data analysis

The focus group data were analyzed according to the six-phase step-by-step guide for thematic analysis outlined by Braun and Clarke [25]. The analysis was an iterative process in which the researchers moved back and forth between the phases. In the first phase, each researcher became familiar with the data by reading and rereading the transcripts to get an overall impression of the findings. One of the first impressions was that although the PHNs were able to identify obesity in children before The First Steps module was introduced, no follow-up tools were available. Implementation of the module improved the ability of the PHNs to focus on diet, weight monitoring, and follow-up.

The next step was to import all transcribed data into the NVivo 12 software package (QSR International, London, UK) for further analysis. In this second phase, the data were organized systematically by open coding using an inductive approach. The corpus data was shortened into smaller units of meanings and coded. Codes were developed inductively and adjusted and collated throughout this phase. In the third phase, the data were organized into more extensive and broader themes until the relationships between codes, themes, and overarching as well as subordinated themes emerged. In the fourth phase, we reviewed the themes to ensure clear and identifiable distinctions between themes. The fifth phase involved defining and naming themes, and in the final phase we produced the report [25]. Throughout the analysis, the data and themes were discussed frequently between researchers until consensus was reached. An illustrative example of the relationships between the corpus data and final themes are given in Table 1.

**Table 1:** Depiction of the relationships between the transcribed data, codes, and one final theme generated through the thematic analysis

Quotes by participants	Code	Theme
*The contact we have before referral [with specialist health-care staff], before they get big … to prevent a referral. Then maybe specialist health-care staff must come to us and give us something concrete. (interview no. 1)*	Gaining competence	The new tools were useful during the follow up of the intricate childhood obesity situation
*We have a tool that can be activated, something to work with, concrete and practical … counselling about diet … I feel more comfortable now than before. (interview no. 1)*		
*What type of yoghurt you choose for instance? Then point and say that you can find that one. Actually, very specific. That’s been good, that I have used a lot. (interview no. 2)*	The information binder is a good tool	
*They got so many eye-openers … was a giant success. When they returned with the boy, they realized that the [weight shown on the] graph was beginning to drop. That was fantastic. (interview no. 2)*		

## Results

### Impact of the first steps routine on overweight and obesity prevention and treatment

The prevalence of overweight including obesity (according to IOTF reference values) at intervention start in 2014 (*n* = 773) was 10.8 and 12.0% among children aged 2–5 years and 6–7 years, respectively. Children measured in 2014 only (*n* = 97) were similar concerning BMI, BMI *z*-scores, age, and the proportion of males/females and normal weight/overweight compared to children measured on both occasions (*n* = 676) (Table 2).

**Table 2:** Descriptive statistics of children included in the study. Numbers are given as mean (SD) unless otherwise stated

	Pre & post cohort (***n*** = 676)	Pre only 2014 (***n*** = 97)	***p***-value^**e**^
**Age in months**^**a**^ **(*min, max*)**	50 (24, 82)	49 (24,84)	0.62
**Boys,** ***n (%)***^**b**^	365 (54)	48 (48)	0.27
**BMI**^**a**^	15.93 (1.51)	16.01 (2.04)	0.67
**BMI** ***z*-score**^**a,c**^	0.11 (1.00)	0.26 (0.83)	0.14
***Underweight (n = 78)***	−1.61 (0.73)	n/a	n/a
***Normal weight (n = 521)***	0.11 (0.55)	n/a	n/a
***Overweight including obesity (n = 77)***	1.81 (0.50)	n/a	n/a
**Overweight including obesity,** ***n (%)***^**a, d**^	77 (11)	10 (10)	0.81
	**2016 minus 2014 values**		
**Change in BMI** ***z*-score**^**c**^	0.09 (0.69)	n/a	n/a
***Underweight (n = 78)***	0.63 (1.01)	n/a	n/a
***Normal weight (n = 521)***	0.06 (0.60)	n/a	n/a
***Overweight including obesity (n = 77)***	−0.26 (0.56)	n/a	n/a

^a^ in 2014; ^b^ Compared to girls, ^c^ according to International Obesity Task Force (IOTF) reference values,

^d^ Compared to normal weight, ^e^ Probability value for differences between children measured in 2014 and 2016 compared to children measured in 2014 only

*Abbreviations*: *n*; numbers, *SD* standard deviation, *BMI* body mass index, *z-score* standard deviation score

Among children measured on both occasions (*n* = 676), during the 2 years, mean BMI *z*-scores increased by 0.63 (95% confidence interval (CI), 0.41, 0.86) for children with underweight and 0.06 (95% CI 0.01, 0.11) in children with normal weight. Further, it decreased by 0.26 (95% CI 0.13, 0.39) in children with overweight or obesity as measured in 2014 (Table 2).

Stratified multiple regression analysis revealed that increased age and baseline BMI *z*-score were associated with and confounded the change in BMI *z*-score for children with under-, normal-, and overweight, whereas sex was not associated with change in BMI *z*-score in any of the groups (Table 3). For children with overweight, results reflect that children who were 1 month older had 0.013 (95% CI 0.007, 0.018) higher change-score (less reduction or increased gain in BMI *z*-score), whereas children had − 0.31 (95% CI -0.54, − 0.09) lower change-score (more reduction or less gain in BMI *z*-score) for each increase by one (1.0) in baseline BMI *z*-score. Age and baseline BMI *z*-score explained 29% of the variance in the mean reduction of BMI *z*-score. The pattern of association was similar for children with normal weight, and similar concerning baseline BMI *z*-score and inverse concerning age in children with underweight. The regression models explained 4 and 40% of the variance in change score for children with normal weight and underweight, respectively.

**Table 3:** Regression coefficients from stratified multiple linear regression analysis associated with change in BMI *z*-score from 2014 to 2016 for children who were underweight, normal weight, and overweight in 2014, respectively

	Unstandardised regression coefficient (95% CI)	Standardized regression coefficient	***p***-value^**d**^	Adjusted R Squared^**e**^
***Stratified to underweight (n = 78)***				0.40
**Constant**	−0.23 (− 0.97, 0.52)		0.55	
**Age in months**^**a**^	**−0.011 (− 0.022, 0.000)**	**−0.19**	**0.04**	
**Boys,** ***n (%)***^**b**^	0.17 (−0.18, 0.53)	0.09	0.38	
**Baseline BMI** ***z*-score**^**a,c**^	**−0.82 (−1.08, − 0.56)**	**−0.57**	**< 0.001**	
***Stratified to normal weight (n = 521)***				0.04
**Constant**	−0.25 (− 0.41, − 0.09)		0.002	
**Age in months**^**a**^	**0.006 (0.003, 0.009)**	**0.18**	**< 0.001**	
**Boys, n (%)**^**b**^	0.06 (−0.04, 0.16)	0.05	0.25	
**Baseline BMI** ***z*-score**^**a,c**^	**−0.13 (− 0.22, − 0.04)**	**−0.12**	**0.01**	
***Stratified to overweight (n = 77)***				0.29
**Constant**	−0.25 (− 0.76, 0.26)		0.34	
**Age in months**^**a**^	**0.013 (0.007, 0.018)**	**0.46**	**< 0.001**	
**Boys,** ***n (%)***^**b**^	−0.12, (− 0.34, 0.10)	− 0.11	0.27	
**Baseline BMI** ***z*-score**^**a,c**^	**−0.31 (− 0.54, − 0.09)**	**−0.27**	**0.01**	

^a^ in 2014; ^b^ Compared to girls, ^c^ according to International Obesity Task Force (IOTF) reference values,

^d^ Probability values for independent variables ≤0.05 are given in bold, ^e^ Explained variance in change of BMI *z*-score from 2014 to 2016 by the models

*Abbreviations*: *n* numbers, *CI* confidence interval, *BMI* body mass index, *z-score* standard deviation score

The prevalence of overweight including obesity (*p* = 0.18) and obesity (*p* = 1.0) did not change significantly in the children measured on both occasions. In 2016, 40 children who were of normal weight in 2014 were classified as overweight or obese, 28 children who were overweight or obese in 2014 were of normal weight, and 49 children were overweight or obese both occasions. Seven children were classified as obese in 2014 but not in 2016 and vice versa, and seven children were obese at both measurement times. Descriptive data of BMI, sex- and age-adjusted BMI *z*-scores according to the IOTF and WHO reference standards, and the prevalence of overweight categories among preschool children aged 2–5 years and > 5 years in the region are presented in supplementary Tables 2 and 3 for all measurements in 2014 and 2016, respectively.

### Experiences of PHNs with the implementation of the first steps module

The PHNs’ experiences of the practical application of The First Steps module were summarized by two themes: 1) The new tools were useful during the follow up of the intricate childhood obesity situation, and 2) Follow-up was like “moving upstream in a river” of complex integrated challenges.

#### Theme 1: the new tools were useful during the follow-up of the intricate childhood obesity situation

The new guidelines were emphasized by the PHNs as a major change in the method for following up children at risk of becoming obese. Before the implementation of The First Steps module, there was no consensus about how to approach families with preschool children identified as being at risk. Parents had mainly been provided with general lifestyle recommendations without further guidance about weight control for their children. The interaction between specialists and primary health-care centers in the municipality related to children with overweight was lacking, and the GP was responsible for referring children for obesity treatment within specialist care. Disagreements between PHNs and GPs about whether a child needed to reduce weight were common. However, after the implementation of The First Steps module, the PHNs noted an increase in the frequency of consultations and weight control efforts.

*We have a tool that can be activated, something to work with, concrete and practical … counseling about diet … I feel more comfortable now than before. (focus group no. 1)*

The information binder with the photographs of healthy food content was useful during the guidance sessions because it gave parents an overview of the recommended food choices. PHNs noted that the binder was helpful in guiding parents of foreign origin and suggested distributing the binder to these parents. According to PHNs, this information was accessible and easier for parents to understand than written texts, which helped them develop new competence.

*There were so many eye-openers … it was a giant success. When they returned with the boy, they realized that the [weight shown on the] graph was beginning to drop. That was fantastic. (focus group no. 2)*

The main improvement perceived by the PHNs was the face-to-face meetings between PHNs and the pediatrician and clinical nutritionist, which provided opportunities for professional discussions. Participants repeatedly emphasized the importance of easy access to the pediatrician and clinical nutritionist. Receiving support about how best to treat a child developing overweight meant that treatment could start early and be delivered within the municipality instead of the specialist health care.

*The contact we have before referral, [with specialist health-care staff] before they get big … to prevent a referral. Then maybe specialist health-care staff can come to us and give us something concrete. (focus group no. 1)*

The module was recommended by all PHNs. They consistently reported positive experiences participating in the project. They reported that the module was consistent with the national guidelines they were required to follow but that it did not significantly increase their workload. Many of the PHNs noted that they had taken a more active role in children’s obesity issues because of the project. The frequent meetings were crucial to the PHNs’ motivation during the project. The PHNs discussed their desire to continue the collaboration with specialist health-care staff as motivation for them to continue providing overweight and obesity prevention and treatment to children.

*We will continue, but we must have someone still pushing us … because I think it is difficult and demanding to pursue. (focus group no. 1)*

Even though the PHNs accentuated how the module’s implementation had led to a more systematic distribution of work assignments between the different professional groups, cooperation with GPs were often described as complicated. Many PHNs reported disagreements with a GP’s approach to parents and children in relation to overweight issues.

*When he [the GP] said that the whole family was big, then it was considered nothing … he did not want to do anything about it … and the mother of course listened to that. (focus group no. 1)*

The PHNs also indicated that there was uncertainty about what kind of role they were supposed to play within the interdisciplinary cooperative approach within primary care services, between primary care and specialist care, and with kindergartens. A clear demand for clarifications of roles and closer cooperation emerged, as did the PHNs’ desire to also use the tools with schoolchildren. They emphasized the need for closer cooperation with kindergartens. Although sufficient collaboration had not become well established, the PHNs generally indicated that they observed an increased focus on healthy diet in kindergartens.

*To get the internal collaboration within the municipality to work … and that parents experience there is like a link … how do kindergartens think, do parents get support from them? Or the opposite? … we must agree. Working alone is difficult if you don’t think similarly. (focus group no. 2)*

#### Theme 2: Follow-up was like “moving upstream in a river” of complex integrated challenges

Given the children’s age, communication with parents was reported as crucial to the PHNs’ ability to guide parents and convey knowledge about a healthy diet. However, the main challenge was to achieve a positive interaction with parents because many parents perceived themselves and their children as vulnerable and dismissive when discussing overweight and obesity issues. Having a child with obesity could create a feeling of failing as a parent.

*Because it is a theme that hits them in a way, they do not want to listen to that. (focus group no. 2)*

*They experience failing as a parent. Many say they feel confronted … that they have not succeeded. (focus group no. 1)*

According to the PHNs’ experiences, most parents were reluctant to talk with their children about issues such as overweight and obesity, and some feared trigging an eating disorder in their child. Parents often normalized and trivialized their child’s weight issues. The focus on weight was reported by PHNs as being overwhelming for some parents, who exhibited a sense of shame and guilt, as well as emotional outbursts such as anger, rejection, anxiety, and crying.

*And being rejected. That’s the actual challenge. And that we have discussed with them [specialist health-care staff]. (focus group no. 2)*

Receiving support, encouragement, and professional guidance from specialist health-care staff and having the courage to work with parents in these difficult situations was frequently discussed in both focus groups. One PHN described counseling sessions with families as “moving upstream in a river.” Several PHNs reported a feeling of powerlessness and that they deliberately avoided mentioning the topic because of resistance from parents. Other problems such as sleeping problems were perceived as more acute and important, and the PHNs often avoided emphasizing weight issues. Parents were perceived as wanting to do their best and the PHNs noted that support must be balanced according the family’s total needs.

*That is actually our profession, that we see the totality. That we see all areas … they don’t manage the day because they have not slept … then we do not talk [about] diet. (focus group no. 1)*

Participants repeatedly noted that childhood obesity has a complex causality. Even though the module provided tools to address overweight issues, the 4-day mapping of the diet was perceived as demanding and the PHNs often doubted the credibility of the parents’ reporting. Despite the parents’ awareness of their child’s obesity, PHNs often felt that parents did not comply with the interventions introduced. Different family composition, culture, and social heritage were highlighted by PHNs. They felt it more burdensome to follow up children of divorced parents who had grater disagreements because the continuity was sometimes a challenge.

*She doesn’t get him [father] on the team, and it is important they agree and work together. That counts not only with diet, but it also counts for everything, and we really see that. (focus group no. 1)*

Particularly in families where the parents themselves were obese, PHNs perceived family needs as more intricate. Often, conflicts between parents about creating firm boundaries for their children were perceived as the actual problem. Participants reported that many parents strived to sustain both their own and their children’s feelings. Food was sometimes used by parents as a means of conflict resolution and consolation. According to the PHNs, disadvantaged families and those with a high level of conflict had a higher risk of developing obesity. PHNs sometimes had to cooperate with child protection services. In such instances, weight issues could provide the entrance to addressing what PHNs perceived as the actual problems.

*Then we may use weight to unravel [the situation] a little. What is going on here? Have things become difficult with boundaries, for instance? (focus group no. 2)*

*There are often also a lot of other things with those families. (focus group no. 1)*

Participants stressed that the whole family had to participate in lifestyle changes to adopt a healthier lifestyle and that the primary responsibility belonged to the parents, particularly when involving preschool children.

*Parents must … they are those who must be treated, it’s not their children. (focus group no. 2)*

Finally, according to the PHNs, the key component for success in reversing the development of obesity was a positive focus on mastery in collaboration with parents.

*They manage … very well actually. The last times now … it’s the opposite, and I can’t get her out of my office. It’s just confirming to her that she is managing. (focus group no. 2)*

## Discussion

The aims of this study were (a) to investigate the impact of The First Steps overweight and obesity prevention and treatment module in routine primary health care including mapping of height/weight and diet followed by parental counselling of healthy habits in children aged 2–7 years, and (b) to explore PHNs’ experiences with the module. The prevention and treatment were based on the national guidelines for primary child health care. Among children measured on both occasions, the proportion of children with overweight and obesity did not differ across the 2 years, whereas the mean BMI *z*-scores increased in children with underweight and normal weight and decreased in children with overweight. The PHNs noted that The First Steps module gave them new tools that were useful for working with the intricate overweight and obesity situation in preschool children. Counseling sessions with families were described as “moving upstream in a river,” and the PHNs noted that overweight including obesity may be one symptom among complex challenges within the families.

### The first steps module’s impact on overweight and obesity

The prevalence of overweight including obesity was slightly lower than the national Norwegian prevalence of 12.7% (2–5 years) and 17.0% (6–11 years) reported in 2010 [6]. However, the prevalence numbers provide a rationale for developing evidence-based and systematic methods to treat and prevent overweight and obesity among young children in the region. To succeed, overweight and obesity treatment in early childhood requires the inclusion of multicomponent interventions that focus on dietary and physical activity behavior and rewards, and modeling should be applied [11, 12].

One may question how national guidelines [17, 18] and The First Steps module align with evidence-based multicomponent interventions. The First Steps module was designed for and applied in child health services. This module focuses on providing support and guidance to parents, as well as support and guidance of PHNs by specialist health-care staff, and focuses mainly on diet. The module is not a standardized multicomponent intervention for treatment of overweight and obesity in children, although the support and guidance included relevant evidence-based components. Therefore, a measurable effect on the proportion of children with overweight including obesity after application of The First Steps module may not have been expected despite the reduction of mean BMI *z*-scores in children with overweight and change in competence and focus among PHNs. We do not know how the competence and focus gained by PHNs from the module was applied in each case. The support and guidance may have varied between individual children and families because these elements were developed through practical applications and adjusted continuously in collaboration between specialist health-care staff and PHNs. Children may also have gained weight, moving from normal weight to overweight, from 2014 to 2016 without having the opportunity to receive any intervention step from the PHNs if they did not have a scheduled follow-up during the same period. Nevertheless, we found that the status of 28 children with overweight including obesity and seven children with obesity in 2014 were normal weight and nonobese in 2016, respectively. This also align with the mean reduction in BMI *z*-scores in children with overweight. The increased impact related to higher baseline BMI *z*-scores may indicate the utility of the module for children with the largest needs. We do not claim this as an effect as documented by a randomized controlled trial but acknowledge the potential that this module had positive health effects in these children, which may continue through their lifespan [26]. While we emphasize the positive impact of the advanced routine child primary health care, we also recognize that several children did not reach a healthier weight. Moreover, increased age at baseline seemed to reduce the impact, confirming the beneficence of early interventions.

### Experiences using the new tools

Our findings from the focus groups with PHNs revealed several aspects, which have been previously reported and synthesized [16]. PHNs’ competence and confidence were changed positively after application of The First Steps module. The feeling of futility, as illustrated by the comment about “moving upstream in a river,” and organizational limitations, particularly those related to the collaboration with the GPs, also align with previously reported experiences [16]. The First Steps module included tools for enhancing parent–professional interaction, understanding of and focus on assessment tools, accommodated language about overweight issues, strengthening of regular routine follow-up, and taking a health-promoting approach when working with the families and kindergartens. Such experiences have also been synthesized and reported as facilitating factors for addressing overweight in children [16].

The PHNs’ experiences of their collaboration with parents were also in accordance with the findings of Bradbury et al. [16] about the challenges in creating a positive collaboration with parents about their child’s weight issues. In the focus groups, the PHNs emphasized gaining insight into, and being able to interact positively with, parents and thereby with the child. Parents may feel unsure about handling their child’s overweight and confused about the PHN’s role [27], which may explain the reported parental emotional outbursts of anger, rejection, anxiety, and crying. The PHNs perceived the support, encouragement, and counseling from specialist health-care staff as giving them the courage to handle rejection by parents and the challenge of motivating both parents to join the team. We recognize that, in addition to negative feelings of parents and their child, the PHNs’ negative feelings, such as the feeling of powerlessness, may be present in counseling situations and may complicate collaboration. As noted in the literature, overweight in parents can create a barrier to treatment of their children [16], and PHNs perceived that communication with these parents was more intricate.

With the support of the module, PHNs could choose which aspects to focus on from what they perceived as the totality of the situation within the context of each family’s challenges, and they could then use this to help unravel what they considered to be the main problem(s). The creation of positive mastery and collaboration with parents was perceived by the PHNs as the key component to dealing with overweight and obesity in children and moving their weight status in a healthier direction. By contrast, the intricacies of the obesity situation and that obesity may be only one symptom among others may explain at least partly why weight status did not change into a healthier category for most of the children.

Our results confirm the complexity of early childhood overweight and obesity treatment in routine health care [11, 12, 16, 27]. Even though The First Steps module provided guidance using evidence-based components and facilitating factors to address overweight and obesity in children, the PHNs experienced challenges to creating mastery and positive collaborations with parents. The PHNs reported challenges relating to different family composition, culture, and social heritage as well as the totality of problems related to parental weight problems, family conflicts, sleeping problems, and creating firm boundaries for children. In a systematic review of nurses’ role and experiences keeping children safe from child abuse and neglect, Lines et al. [28] reported the need for increased knowledge, communication, and validation to put the “pieces of a jigsaw-puzzle” together, as well as challenge of balancing surveillance with support. The PHNs’ experiences discussed in the focus groups in the current study indicate that overweight and obesity prevention and treatment requires a similar approach. Our findings suggest that routine health care for childhood overweight and obesity may overlap with the concept of the totality of the child and family problems and that routine child follow-up by PHNs should be improved. Preventing unhealthy weight development in children in the context of multiple childhood and family problems will require integration of research and health-care practice that involves placing overweight issues within the totality of the child’s and family situation. A more systematic approach to mapping and treatment, as well as integration of professional guidance from specialists into routine follow-up may provide a promising pathway for both areas of child and family problems.

### Study strengths and limitations

The strengths of the current study are the inclusion of both cross-sectional and longitudinal sex- and age-adjusted weight and height data from the same time period as the practical application of the module and the focus group data concerning PHNs’ experiences with this module. Reciprocal reflection between researchers about the analysis and findings of both data sources strengthen the trustworthiness of the insights gained. The study was limited by a lack of direct link between the use of the module and each child and family who received health care, as well as the lack of a strictly standardized intervention and randomization of the children. The follow-up time of 2 years may have been too short to show a change in the proportion of children with overweight and obesity. The study also did not include information about parental experiences. Readers should be aware that the issues reported from the focus group data rely entirely on the PHNs’ experiences, which may have differed from those of the parents and children.

## Conclusion

The prevalence of overweight and obesity in 2014 was 10.8 and 12.0% among children aged 2–5 years and > 5 years, respectively. The mean BMI *z*-scores decreased during the 2-year implementation of the stepwise overweight and obesity treatment module among overweight children, whereas the proportion of children with overweight including obesity was stable. PHNs perceived that the module provided new and useful tools for approaching overweight and obesity among preschool children and that such work is intricate. They described the collaboration with families for overweight and obesity treatment as “moving upstream in a river” and that overweight and obesity may be one symptom among complex challenges within families. PHNs considered the collaboration with specialist health-care staff necessary for delivering this overweight and obesity prevention module.

The main implication of the current findings is that specialist- and evidence-based support is needed to ensure effective prevention and treatment of childhood overweight and obesity in primary health care. Mapping of issues related to overweight and other child and family problems in both clinical practice and research is also needed.

## Supplementary Information

**Additional file 1: Table S1.** Descriptive weight data from measurements for the 2014 cohort (*n* = 773). **Table S2.** Descriptive weight data from measurements for the 2016 cohort (*n* = 919)

**Additional file 2:.** Focus group guide for focus group interviews with public health nurses.

## Data Availability

The datasets generated and/or analyzed during the current study are not publicly available due to recommendations by the Norwegian Social Science Data Service, but are available from the corresponding author on reasonable request.

## Abbreviations

BMI: Body mass index
CI: Confidence interval
GP: General practitioner
IOTF: International Obesity Task Force
PHN: Public health nurse
WHO: World Health Organization
