# Supplementary material for: Prevention of overweight and obesity in a Norwegian public health care context: a mixed-methods study
Source: BMC Public Health. 2021 May 26;21:983. doi: 10.1186/s12889-021-11096-x (PMC8152087; doi:10.1186/s12889-021-11096-x)
Supplement: Supplementary file 1 — Additional file 1: Table S1. Descriptive weight data from measurements for the 2014 cohort (n = 773). Table S2. Descriptive weight data from measurements for the 2016 cohort (n = 919) [file 12889_2021_11096_MOESM1_ESM.docx]

**Supplementary Table 1.** Descriptive weight data from measurements for the 2014 cohort (*n* = 773)

|  | **BMI, mean (SD)** | **IOTF BMI SDS, mean (SD)** | **WHO BMI SDS, mean (SD)** | **IOTF category, *n* (%)** | | | **WHO category, *n* (%)** | | |
| --- | --- | --- | --- | --- | --- | --- | --- | --- | --- |
|  |  |  |  | **OWOB** | **OB** | **VOB** | **OWOB** | **OB** | **VOB** |
| **2–5 years (*n* = 499)** |  |  |  | 54 (10.8) | 9 (1.8) | 2 (0.4) | 23 (4.6) | 4 (0.8) | - |
| **2–5 years boys (*n* = 268)** | 16.28 (1.44) | 0.14 (1.02) | 0.46 (1.00) | 31 (11.5) | 6 (2.2) | - | 14 (5.2) | 2 (0.7) | - |
| **2–5 years girls (*n* = 231)** | 15.94 (1.43) | 0.04 (0.99) | 0.30 (0.95) | 23 (9.9) | 3 (1.3) | 2 (0.9) | 9 (3.9) | 2 (0.9) | - |
| **>5 years (*n* = 274)** |  |  |  | 33 (12.0) | 6 (2.2) | 3 (1.1) | 43 (15.7) | 12 (4.4) | 3 (1.1) |
| **>5 years boys (*n* = 142)** | 15.70 (1.48) | 0.12 (0.95) | 0.20 (1.00) | 14 (9.7) | 3 (2.1) | 2 (1.4) | 23 (16.2) | 6 (4.2) | 2 (1.4) |
| **>5 years girls (*n* = 132)** | 15.71 (1.59) | 0.24 (0.94) | 0.19 (0.92) | 19 (14.4) | 3 (2.3) | 1 (0.8) | 20 (15.2) | 6 (4.5) | 1 (0.8) |

Abbreviations: *n*; number, BMI; body mass index, SD; standard deviation, SDS; standard deviation score (z-score), WHO; World Health Organization, IOTF; International Obesity Task Force; OWOB; overweight including obesity, OB; obese, VOB; very obese

**Supplementary Table 2.** Descriptive weight data from measurements for the 2016 cohort (*n* = 919)

|  | **BMI, mean (SD)** | **IOTF BMI SDS, mean (SD)** | **WHO BMI SDS, mean (SD)** | **IOTF category, *n* (%)** | | | **WHO category, *n* (%)** | | |
| --- | --- | --- | --- | --- | --- | --- | --- | --- | --- |
|  |  |  |  | **OWOB** | **OB** | **VOB** | **OWOB** | **OB** | **VOB** |
| **2–5 years (*n* = 423)** |  |  |  | 43 (10.2) | 5 (1.2) | 2 (0.5) | 19 (4.5) | 2 (0.5) | - |
| **2–5 years boys (*n*= 231)** | 16.37 (1.38) | 0.20 (0.98) | 0.52 (0.98) | 23 (10.0) | 4 (1.7) | 1 (0.4) | 13 (5.6) | 1 (0.4) | - |
| **2–5 years girls (*n* = 192)** | 16.05 (1.31) | 0.12 (0.92) | 0.38 (0.88) | 20 (10.4) | 1 (0.5) | 1 (0.5) | 6 (3.1) | 1 (0.5) | - |
| **>5 years (*n* = 496)** |  |  |  | 68 (13.7) | 11 (2.2) | 2 (0.4) | 100 (20.2) | 28 (5.6) | 2 (0.4) |
| **>5 years boys (*n*= 264)** | 16.19 (1.96) | 0.22 (0.98) | 0.27 (1.09) | 38 (14.4) | 6 (2.3) | 1 (0.4) | 61 (23.1) | 17 (6.4) | 1 (0.4) |
| **>5 years girls (*n* = 232)** | 15.95 (2.11) | 0.13 (0.98) | 0.08 (0.99) | 30 (12.9) | 5 (2.1) | 1 (0.4) | 39 (16.8) | 11 (4.7) | 1 (0.4) |

Abbreviations: *n*; number, BMI; body mass index, SD; standard deviation, SDS; standard deviation score (z-score), WHO; World Health Organization, IOTF; International Obesity Task Force, OWOB; overweight including obesity, OB; obese, VOB; very obese
