# Supplementary material for: Prevention of overweight and obesity in a Norwegian public health care context: a mixed-methods study
Source: BMC Public Health. 2021 May 26;21:983. doi: 10.1186/s12889-021-11096-x (PMC8152087; doi:10.1186/s12889-021-11096-x)
Supplement: Supplementary file 2 — Additional file 2:. Focus group guide for focus group interviews with public health nurses. [file 12889_2021_11096_MOESM2_ESM.docx]

The first steps - Targeted follow-up of children 0-6 years to prevent obesity.

**Focus group guide for focus group interviews with public health nurses**

**Introduction**

- Presentation of the moderator and co-moderator
  - Clarify the moderator and co-moderator’s role.
- Brief presentation of the qualitative study and the focus group.
- Ensure that participants are informed / have signed the consent form.
- Information about tape recording and deletion of this.
- Encourage mutual confidentiality concerning information shared in the focus group.
- Ask if there is anything the participants are wondering about / questions before we start.

**Experiences in relation to the use of tools in to the 3-step module for overweight**

- How did you identify children 0-6 years old with a high risk of obesity before you started using action cards?
- How has the use of the stepwise tools affected the way you as a public health nurse identify and follow up children 0-6 years with a risk of obesity?
  - What has worked well regarding the use of the tools?
  - Has there been any challenges associated with the use of tools? (please explain)
- Can you tell us about a case where you think that the use of the stepwise tools worked well? Did not work well?
  - Tell us about a situation where a child had to proceed to step 1, 2 or 3 - how did you do this?
  - Tell us about how your identification and follow up these children has changed.
- Is there anything you think could have been set up differently to improve the use of stepwise tools? (explain the answer)

**Interaction between the primary health care services and specialist health care services**

- What is your experience of how the use of the stepwise tools affects the collaboration on follow-up of overweight children between the primary- and the specialist health care services?
  - What has been working well in relation to the collaboration?
  - What collaboration challenges do you experience concerning the use of the tools?
- If you could choose, is there anything you think could have been done differently to improve this collaboration? (explain the answer)

**Closure**

- Is there anything further you would like to tell us that could be useful information in relation to this study?
- Would you recommend other public health nurses to use the tools according to the 3-step module? (explain the answer)
- Repetition of information given before the focus group.
